# Advancing Agricultural Production With Machine Learning Analytics: Yield Determinants for California’s Almond Orchards

**DOI:** 10.3389/fpls.2020.00290

**Published:** 2020-03-13

**Authors:** Yufang Jin, Bin Chen, Bruce D. Lampinen, Patrick H. Brown

**Affiliations:** ^1^Department of Land, Air and Water Resources, University of California, Davis, Davis, CA, United States; ^2^Department of Plant Sciences, University of California, Davis, Davis, CA, United States

**Keywords:** *Prunus dulcis*, yield gap, artificial intelligence, big data, light interceptioon, nutrient management

## Abstract

Agricultural productivity is subject to various stressors, including abiotic and biotic threats, many of which are exacerbated by a changing climate, thereby affecting long-term sustainability. The productivity of tree crops such as almond orchards, is particularly complex. To understand and mitigate these threats requires a collection of multi-layer large data sets, and advanced analytics is also critical to integrate these highly heterogeneous datasets to generate insights about the key constraints on the yields at tree and field scales. Here we used a machine learning approach to investigate the determinants of almond yield variation in California’s almond orchards, based on a unique 10-year dataset of field measurements of light interception and almond yield along with meteorological data. We found that overall the maximum almond yield was highly dependent on light interception, e.g., with each one percent increase in light interception resulting in an increase of 57.9 lbs/acre in the potential yield. Light interception was highest for mature sites with higher long term mean spring incoming solar radiation (SRAD), and lowest for younger orchards when March maximum temperature was lower than 19°C. However, at any given level of light interception, actual yield often falls significantly below full yield potential, driven mostly by tree age, temperature profiles in June and winter, summer mean daily maximum vapor pressure deficit (VPD_max_), and SRAD. Utilizing a full random forest model, 82% (±1%) of yield variation could be explained when using a sixfold cross validation, with a RMSE of 480 ± 9 lbs/acre. When excluding light interception from the predictors, overall orchard characteristics (such as age, location, and tree density) and inclusive meteorological variables could still explain 78% of yield variation. The model analysis also showed that warmer winter conditions often limited mature orchards from reaching maximum yield potential and summer VPD_max_ beyond 40 hPa significantly limited the yield. Our findings through the machine learning approach improved our understanding of the complex interaction between climate, canopy light interception, and almond nut production, and demonstrated a relatively robust predictability of almond yield. This will ultimately benefit data-driven climate adaptation and orchard nutrient management approaches.

## Introduction

Global food and fiber demand has been projected to double by the mid-century, driven mostly by increasing population and nutrition needs (Tilman et al., 2011; Davis et al., 2013). However, agricultural production has been shown vulnerable to multiple stresses including warming, droughts and floods, extreme weather variability (Rosenzweig et al., 2001; Reynolds and Tuberosa, 2008; Funk and Brown, 2009; Lesk et al., 2016), and degrading soils and water (Elliott et al., 2014). Growers face the grand challenges of increasing food production while minimizing environmental disruption, and improving the resilience of agriculture systems under changing climates (National Academies of Sciences Engineering Medicine, 2019). Optimizing food system requires a new approach that integrates existing datasets for new insights about yield determinants, and resolves the complex and interconnected physical and biological processes affecting yield across different scales. Recent technological advances in artificial intelligence provide promising tools to understand the constraints on potential yield and interpret and predict the variation of yield across space and time by harnessing many unique yet under-utilized datasets.

California’s almond acreage has expanded rapidly in recent decades, from 283,280 hectares in 2005 to 538,232 hectares in 2017 (USDA-NASS, 2018), due to the increasing demand for almonds in domestic and international markets. Almond has become the second leading agricultural commodity in California, with a total farm gate value of 5.6 billion US dollars in 2017 (California Department of Food and Agriculture, 2017). California produces about 80% of the world’s almonds and 100% of the U.S. commercial almond production. More than 95% of almond acreage is irrigated and growers rely heavily on surface irrigation deliveries and on groundwater when surface water is limited, as occured during the recent prolonged 2013–2017 drought in California (Faunt et al., 2016). Climate change, including warming and extreme weather, is another threat to almond production. The projected climatic conditions by the middle to end of the 21st century are predicted to threaten the long-term viability of the state’s almond production (Luedeling et al., 2009). To optimize yield and ensure the almond industry remains economically viable and environmentally sustainable (Carletto et al., 2015; Tombesi et al., 2017), it is essential to understand key yield determinants and develop appropriate agricultural adaptation and management strategies.

Groundwater quality in California has also been degraded due to nitrogen leaching from agricultural fields (Burow et al., 2013; Baram et al., 2016). Facing with this serious challenge, the state of California has implemented legislatively mandated nitrogen (N) management strategies for all almond growers statewide to meet the goal of minimizing nitrogen losses to the environment. To optimize N management and ensure regulatory compliance, almond growers must now apply N in accordance with the estimated yield determined in each orchard in early spring, taking into account N available from all sources (e.g., fertilizer, composts and manures, and irrigation water nitrogen). Accurate yield prediction is thus critically important to help individual growers with the information required to manage inputs and resources, to schedule on-farm activities and manage harvest and marketing agreements.

Almond yield varies by year and by location; however, the environmental and biophysical factors that underlie these differences are not well understood and have never been systematically characterized. Almond production is known to be highly dependent on a number of factors (Tombesi et al., 2010, 2017; Zarate-Valdez et al., 2015) including (a) biophysical attributes such as tree age, leaf area, tree vigor, and bloom intensity, (b) environmental conditions such as chilling and heat requirements, soil nutrition, and bee foraging activity, and (c) cropping history. To date, a detailed comprehensive assessment of each of these factors and a yield prediction algorithm has not been successfully achieved, especially at a finer spatial scale.

Among the variables that have been shown to impact yield in almond, canopy interception of photosynthetically active radiation (PAR), is directly related to maximum potential yield of almonds (Zarate-Valdez et al., 2015). Lampinen et al. (2012), reported that the maximum sustainable yield in the most productive commercial almond orchards is 56 kernel kg/ha per unit PAR intercepted by the canopy. Percent light interception at the orchard level is determined by canopy structure, e.g., total leaf area and health at the individual tree level, as well as row and tree spacing; while the location of the orchard (latitude) and cloud fraction affect the total amount of PAR incident on the canopy. Management activities such as cultivar selection, tree spacing, pruning practices, nutrition, and irrigation also have direct impacts on canopy interception and thus yield. As almond is a perennial crop, the multi-year photosynthetic accumulation and allocation to reproductive and vegetative organs from previous years also affect its yield (carry over effect), as well as spurs frequency.

Climate, such as temperature and water availability, is known to have an important role in crop growth and flowering, and thus influencing yield variation (Kerr et al., 2018; Pathak et al., 2018). A few prior studies have used relatively simple statistical analysis to understand how temperature and precipitation affected almond yield in California, but were largely limited by the spatial scale, e.g., from county to state levels (Lobell et al., 2007; Lobell and Field, 2011), and temporal coverage, resulting relatively small sample size for analysis (e.g., from tens to hundreds). At the scale of an individual plant, growth models developed by DeJong (2019) as well as knowledge of the role of flower number on yield potential (Tombesi et al., 2017) and modeled carbon budgets all contribute knowledge that can be integrated into a yield prediction model. However, these mechanistic approaches have not been systematically applied at any significant scale.

Moreover, nut production of almond trees is also highly dependent on bee pollination. Most almond cultivars are self-sterile, and two or more cultivars are usually inter-planted (Connell, 2000). Bee foraging activity is thus a crucial determinant of the final yield. In addition to being dependent on environmental variables such as temperature, solar radiation, and wind, bee activity is highly reliant on the timing and intensity of flowering, which in turn is also highly affected by weather conditions. Understanding these complicated impacts of environmental factors on almond nut production is therefore rather challenging, especially at the individual field level, requiring a large spatial and temporal data set and more advanced analytical algorithms.

To address these issues and develop a yield prediction model and descriptor of key yield determinants on almond, we have obtained a 10-year collection of plant and field level biological measurements, management practices, and yield records from 33 locations across the main growing regions of California. Using an advanced machine learning algorithm, we integrated these data with two meteorological datasets to investigate the environmental, biological, and management factors that determine yield variability of almond. Specifically, we aim to answer the following scientific questions: (i) what are the limiting factors that affect yield at a given level of light interception? (ii) Is it possible to predict light interception with orchard age and environmental variables? and (iii) What are the overall impacts of environmental variables on actual yield when controlling for both light interception and the yield gap at a given light interception? An improved understanding of these questions is expected to guide and optimize the life-cycle management of almond production. There is considerable commercial interest in the ability to predict yield and identify production constraints effectively and, as a consequence, the models and information developed in this paper will also be useful to optimize management and hence sustainability.

## Materials and Methods

### Study Area

**FIGURE 1:**
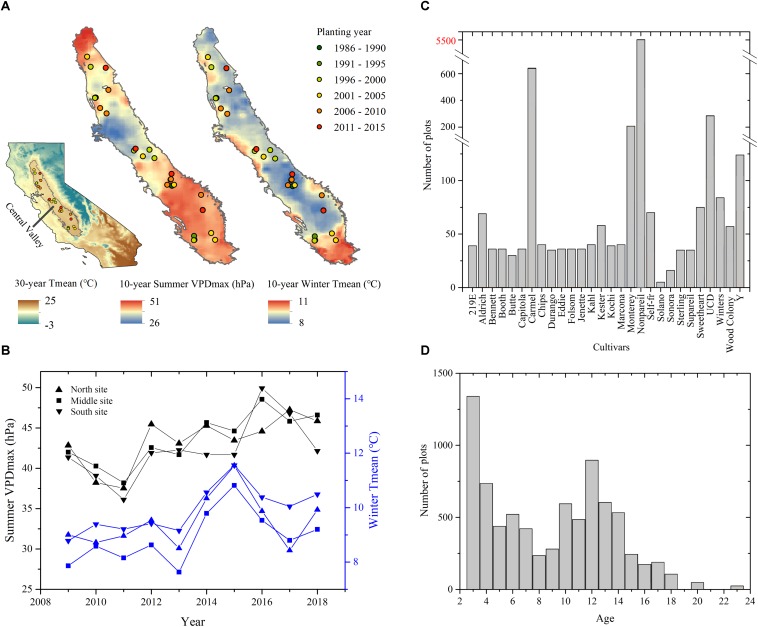
Study sites of almond orchards in California’s Central Valley. **(A)** Orchard locations overlaid on the mean summer daily maximum Vapor Pressure Deficit (VPD_max_) and mean winter Temperature (T_mean_) averaged during 2009–2018. The inset shows the extent of Central Valley in the state of California overlaid on the 30-year mean monthly temperature during 1980–2010. **(B)** Annual time series of mean summer daily VPD_max_ and winter T_mean_ averaged over northern, middle, and southern study sites from 2009 to 2018. Also shown are the distributions of **(C)** cultivars and **(D)** orchard age for all plots at the sampling years.

Our study area focused on California’s Central Valley, one of the most productive agricultural areas in the world. We have a 10-year collection of field measurements and yield records over a total of 33 individual almond orchards containing 7865 individual experimental plots (Figure 1). This region experiences a Mediterranean climate characterized by hot and dry summers and mild and wet winters. Typically, the rainless summer provides ample sunshine for almond growth and limits disease pressures. The cool and wet winter replenishes the soils and reservoirs in bordering mountainous areas, this and groundwater resources provide water for irrigation during the dry season.

### Field Measurements

We collected canopy light interception and yield data over 33 almond orchards, that included a total of 7864 experimental plots, spanning the almond producing areas of the Sacramento and San Joaquin valleys of California, from 2009 to 2018 (Lampinen et al., 2012). The consistent practice of sample collection, supported by Almond Board of California, was designed to evaluate and understand almond production characteristics and drivers from a single tree to orchard scale, for the purpose of improving almond orchard management. For each plot, trees were randomly sampled over a full row length ranging from 50 to 150 individuals for canopy light interception measurement during May to August growing season. A mobile platform (MLB hereafter), consisting of a series of 18 ceptometer segments mounted on a Kawasaki mule utility vehicle, was used to measure PAR below the canopy of both sides of almond trees (*PAR*_*below*_). Simultaneously, a fixed light sensor recorded the full sun incoming PAR above the canopy (*PAR*_*above*_). All PAR measurements were conducted at solar noon (±1 h), and the light interception was calculated as the fractional PAR intercepted by the canopy:

(1) $$L\,R=f\,P\,A\,R=1-\frac{P\,A\,R_{b\,e\,l\,o\,w}}{P\,A\,R_{a\,b\,o\,v\,e}}$$

For each individual experimental plot, average fPAR values of individual trees were calculated to represent the plot-level light interception.

Almond trees were harvested by shaking with a mechanical shaker and the nuts were collected after letting them dry on the ground for about 1 week. Fresh fruit weight was recorded for each individual experimental tree, and a 2 kg sample was used for dry fruit weight (hull plus shell plus kernel) and dry kernel yield (i.e., the yield value used in this study). For each experimental plot, we also recorded its specific orchard site, geographic location (latitude and longitude), planting year, cultivar composition, row and tree spacing (Table 1).

**TABLE 1:** Summary of input variables in this study.

Data source	Input variables	Variable name	Pearson’s *r* (***p < 0.001)	
**Biological variables of almond orchards**				
Field measurement	Latitude	Lat	−0.17***	
	Longitude	Lon	0.16***	
	Cultivar	Cul	−0.15***	
	Tree age	Age	0.46***	
	Row spacing	Row	0.10***	
	Tree spacing	Tree	0.10***	
	Light interception	LI	0.60***	

**Meteorological variables (averages over 12 individual months and 4 seasons from daily values)**				

**PRISM (4 km)**	**(http://www.prism.oregonstate.edu/)**		**Current year**	**Previous year**

	Precipitation	PPT	−0.26 ∼0.20	−0.19 ∼0.05
	Maximum temperature	T_max_^1^	−0.15 ∼0.09	−0.05 ∼0.19
	Minimum temperature	T_*min*_^1^	−0.24 ∼0.19	0.07 ∼ 0.20
	Mean temperature	T_mean_^1^	−0.19 ∼ 0.16	0.01 ∼ 0.20
	Maximum vapor pressure deficit	VPD_max_^1^	−0.30 ∼0.06	−0.17 ∼0.08

**Daymet (1 km)**	**(https://daymet.ornl.gov/)**			

	Daylight duration	Dayl	0.08 ∼ 0.20	−0.21 ∼0.17
	Shortwave radiation flux density	SRAD	0.05 ∼ 0.24	−0.04 ∼0.30
	Extremely hot days	HotDays	−0.06 ∼−0.25	−0.09 ∼−0.19

*The Pearson’s correlation and its significance between each individual variable and the production were shown here. For time varying meteorological variables, the range of the statistics for each variable among monthly and seasonal parameters were included. ^1^Monthly and seasonal variables were mean values of daily maximum, minimum, and mean averaged over each month or season.*

### Climate and Weather Data

We used monthly climate record from the Parameter-Elevation Regressions on Independent Slope Model (PRISM) dataset (Daly et al., 2008), including monthly mean values of daily precipitation, daily maximum/minimum/mean temperature, and daily maximum VPD (VPD_max_) (Table 1). PRISM uses weather station observations, a digital elevation model (DEM), and other spatial datasets to extrapolate the observations from weather stations to ∼ 4-km gridded estimates of monthly climatic variables over the United States (Daly et al., 2008, 2015).

We used the daily weather data at 1km scale from the Daymet Version 3 product, to quantify incoming shortwave radiation flux density (SRAD) at the surface and the duration of the daylight period (Dayl) (Thornton et al., 2017). We further derived the total number of extreme hot days for each month (HotDays). For each month, the threshold of daily T_max_ was set as the upper 10-percentile daily maximum temperature from 2009 to 2018, respectively, based on the daily DayMet T_max_ product. If the daily T_max_ for a certain day exceeded the extreme threshold value of the corresponding month, it was identified as a relatively hot day. All the monthly variables (except for Hotdays) from 2009 to 2018 were further aggregated to derive 10-year mean climatology at both seasonal (i.e., spring, summer, fall, and winter) and annual scales. Climate from both current year and preceding years were also explored for our analysis.

### Yield Potential

Higher light interceptions usually lead to higher yields, but the yield also varies significantly with other environmental stressors (Lobell et al., 2007; Tombesi et al., 2010; Zhang et al., 2019). To understand the maximum yield potential that almond could reach at a given light interception, we grouped all plot-year samples by the associated light interception with an interval of 5%, and selected the upper 10-percentile samples within each light interception bin, as a proxy for the yield potential. The light interception and its corresponding yield were then averaged over the subsamples for each group to model the upper bound of the yield at a given light interception percentage. A linear regression model was built with the interception set to zero. We conducted this analysis for all plots (*n* = 7864), and for a subset of plots (*n* = 5581) containing the most dominant cultivar, Non-pareil, respectively.

### Environmental Stressors for Yield Gap

To further understand the factors that constrained the almond trees from reaching the maximum yield under a given level of light interception, we normalized the original yield by the modeled yield potentials, as follows:

(2) $$y_{n}=\frac{y_{o}}{y_{p}}$$

where *y*_*o*_ is the original yield, *y*_*p*_ is the modeled yield potential, and *y*_*n*_ is the final normalized yield, typically ranging from 0 to 1 (with very few samples beyond 1). Samples with *y*_*n*_ less than 1 indicated productivity under the yield potential. The deviation of *y*_*n*_ from 1 can therefore be used as a proxy for the yield gap.

We used the random forest machine learning approach to model and analyze the complex relationship between the normalized yield and a suite of meteorological variables, in order to understand what and how environmental stressors limit the yield at given light interception. Random forest is an ensemble learning technique to improve classification and regression trees method by combining a large set of decision trees (Liaw and Wiener, 2002; Belgiu and Drăgut̨, 2016; Jeong et al., 2016). In random forest regression, each tree is built using a deterministic algorithm by selecting a random set of variables and a random sample from the training dataset. Specifically, the “RandomForest” package within R environment software was used in this study^1^.

Conceptually monthly and seasonal meteorological variables, during both the current year and the preceding year, may pose stresses atthe different stages of plant growth, including flowering, leaf out, and fruit setting (Tombesi et al., 2010, 2017). Although a large set of explanatory variables is not an obstacle for the functioning of random forest model, the highly correlated meteorological variables may hinder the interpretation of the modeling results (Liaw and Wiener, 2002). We first used Pearson’ correlation coefficient (r) to investigate how each individual independent variable was correlated with the yield gap, and how each individual weather variable correlated with each other among different time periods, thereby providing the basis for selecting a subset of more significant meteorological variables for building the model. In this study, we selected representative variables that are highly correlated with yield gap (i.e., *r* > 0.15) and less cross-correlated with other variables within the same category (i.e., *r* < 0.50) (Supplementary Figures S1, S2 and Supplementary Table S1).

With random forest modeling, we ranked the variable importance based on how much the modeling accuracy decreased, or the increase in mean-square-error (i.e., IncMSE) of predictions, when a particular variable was excluded from the whole suite of input variables for model building (Grömping, 2009). The IncMSE of predictions, estimated with an out-of-bag cross validation, in percentage relative to the full model, is a robust and informative metric, e.g., higher values indicating that the corresponding variable is more important for yield prediction.

We further used partial dependence plots to understand how each of these variables affected the yield (Welling et al., 2016). Intuitively, partial dependence plots show the dependence between the target response and a set of explanatory features, marginalizing over the values of all other features. We can interpret the partial dependence as the expected target response as a function of the explanatory features.

To further examine what conditions or combinations of conditions are associated with relatively higher or much lower normalized yield, we used the regression tree model (i.e., “rpart” package within R environment^2^) to identify decision rules between explanatory variables and the target response that can best differentiate yield gaps, i.e., representative splitting nodes. We chose the decision tree with a highest predictive accuracy as the most representative tree in this study.

### Determinants of Light Interception

As a dominant influential variable, light interception (or percentage of absorbed PAR) reflected the combined effects of canopy density, structure, and health status, which were again associated with tree age, row and tree spacing at a plot level, and meteorological conditions that affected tree physiology and development (Zarate-Valdez et al., 2015). To understand the dominant factors that affected light interception, we also analyzed the relationship between the light interception percentage and a suite of layers (including orchard characteristics, and current and preceding meteorological variables), using random forest model. Non-pareil was used as an example for this analysis (*n* = 5581), to exclude the potential confounding factors from different cultivars.

### Drivers for Overall Almond Yield

Besides affecting the light interception via tree growth and health, environmental variables may also affect flower phenology, bee activities, pollination, fruit set, and production. To further examine the complex relationships between yield and biological and environmental controls, we built overall random forest models to predict almond yield at the plot level, driven by four sets of independent variables, respectively. Specifically, these included (A) biological variables including measured light interception percentage and cultivar composition (Table 1), (B) biological variables and full meteorological variables (Supplementary Figure S4), (C) biological variables and selected meteorological variables, and (D) biological variables but excluding light interception and full meteorological variables. Model performance was evaluated and compared with a sixfold cross validation. The root mean square error (RMSE) and R-square (*R*^2^) were used to quantify the models’ accuracy. We also calculated a ratio of performance to interquartile distance (RPIQ), which accounts for both the prediction error and variation of observed values, and therefore it is more objective than the RMSE and easier to compare among models (Bellon-Maurel et al., 2010). A greater RPIQ represents a stronger predictive capacity of the model (Bellon-Maurel et al., 2010).

## Results

### Controls on Almond Yield Potential

**FIGURE 2:**
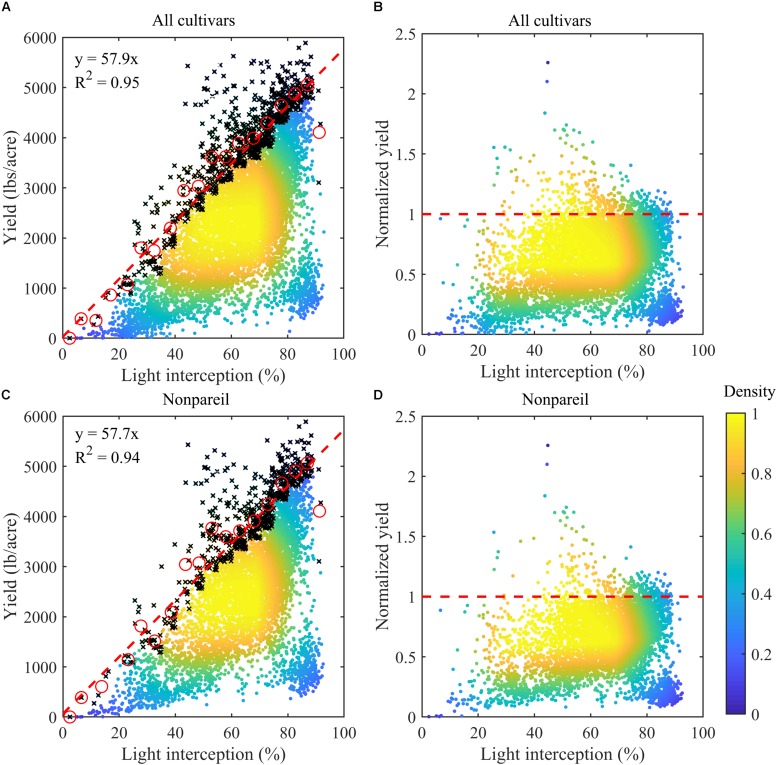
Almond yield vs. light interception percentages (LI) for **(A)** all samples (*n* = 7864) and **(C)** Non-pareil samples (*n* = 5581) in the experimental plots. Color represents the density of the samples. The mean values (red circles) of the upper 10-percentile samples (black crosses) within each 5% of LI interval were used for modeling the potential yield, with regression lines shown in red dashed lines in **(A,C)**. Also shown are the corresponding normalized yields (actual yield divided by the corresponding modeled yield potential) for **(B)** all cultivars and for **(D)** Non-pareil.

Overall almond yield highly depended on light interception (Figure 2A), as shown by the Pearson’s correlation coefficient of 0.60 (*p* < 0.001) between the recorded yield and measured light interception percentage across all sample plots. Yield increased from 467.4 ± 432.6 lbs/acre to above 2907.6 ± 1084.2 lbs/acre, when LI increased from below 30% to above 70%. Across each 5% interval of light interception, we found a very strong linear relationship between the maximum yield, as represented by the upper 10-percentile samples, and the light interception (Figures 2A,C). The yield potential predicted by the linear regression model agreed well with the observation, with a *R*^2^ of 0.95, when all cultivars were considered. In particular, we found that one percent of increase in light interception led to an increase of 57.9 lbs/acre in the potential yield, as shown by the slope of the regression model (Figure 2A). Similar results were found when the analysis was restricted to the cultivar Non-pareil (*R*^2^ = 0.94, slope = 57.7 lbs/acre per LI unit), further supporting that the yield potential was dominated by the light interception (Figure 2C).

### Determinants on Almond Yield Gap

Actual almond nut production was found to vary significantly at a given level of light interception (Figure 2A), even for the same cultivar (Figure 2C). For example, Non-pareil trees had yields ranging from 2278 lbs/acre (lower quantile) to 3267 lbs/acre (upper quantile), and averaged 2790 ± 781 lbs/acre, when LI was between 70% to 75% (Figure 2C). Across all plots the majority of almond samples didn’t reach yield potential (i.e., red dashed line) for any given light interception percentage (Figures 2B,D).

**FIGURE 3:**
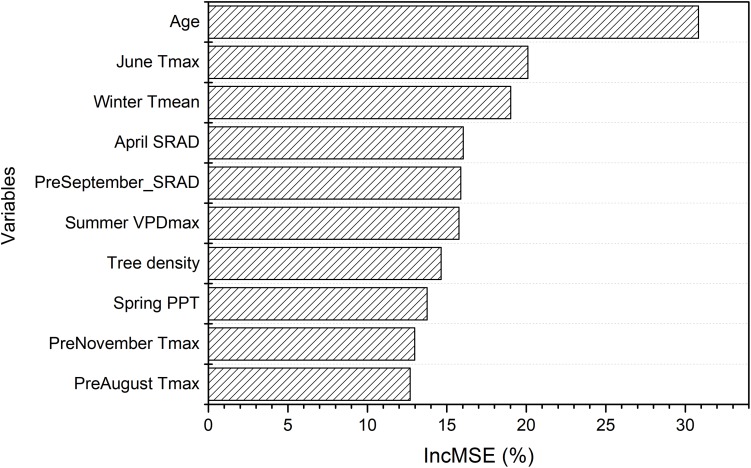
Variable importance from the random forest model of yield gap, as measured by the increase in mean-square-error (IncMSE) of predictions when excluding each variable.

**FIGURE 4:**
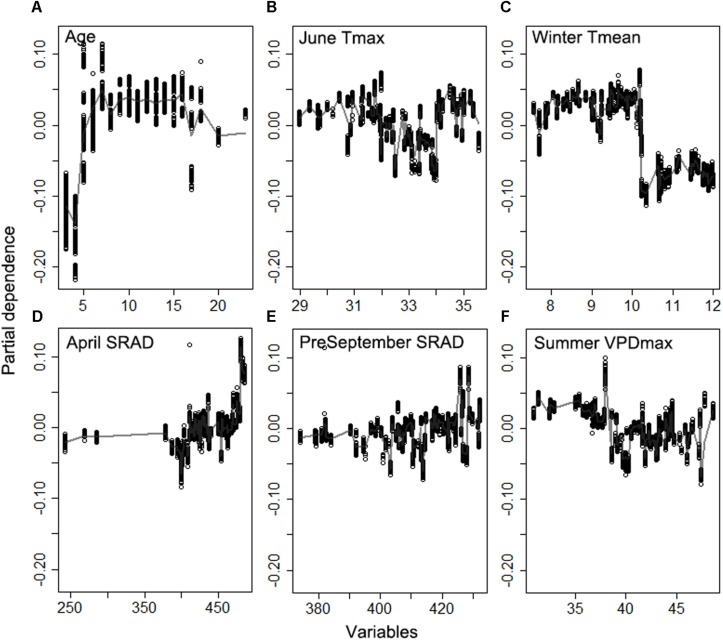
Partial dependence of the normalized yield (actual yield divided by modeled yield potential) on the top six important variables **(A)** Age, **(B)** daily maximum temperature averaged over June, **(C)** daily mean temperature averaged over winter, **(D)** daily shortwave radiation flux density (SRAD) averaged over April, **(E)** daily SRAD averaged over previous September, **(F)** daily maximum vapor pressure deficit (VPD) averaged over summer for Non-pareil, ordered by variable importance.

The random forest analysis, as described in section “Yield Potential,” showed that the variation of yield gap, 1- actual yield normalized by the potential yield at the corresponding light interception, was mostly driven by tree age, mean June daily T_max_, winter T_mean_, SRAD, and mean summer daily VPD_max_, among orchard characteristics and climate variables (Figure 3). Mature orchards (>5 years old) tended to have lower yield gap than younger orchards for the same amount of light interception and climate (Figure 4A). The partial dependence plots also showed that almond yield dropped significantly below the yield potential when the average winter temperature was higher than 10°C and April SRAD was lower than 450 W m^–2^ (Figures 4C,D). Daily T_max_ averaged in June, daily SRAD averaged over previous September, and daily VPD_max_ averaged in summer had a more gradual impact rather than a significant thresholding effect (Figures 4B,E,F).

**FIGURE 5:**
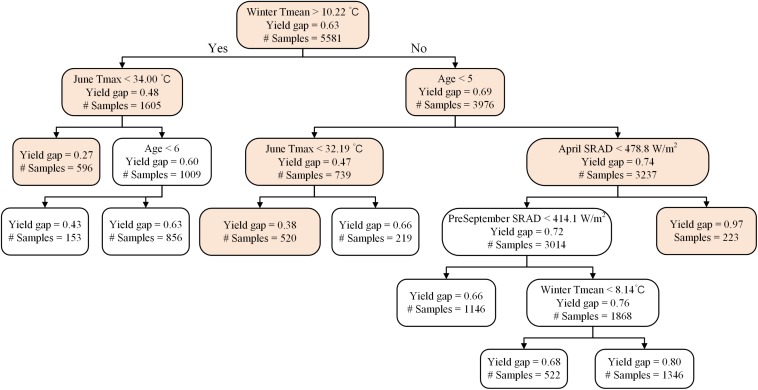
A representative decision tree on the drivers of actual yield gaps (actual yield divided by modeled yield potential) for Non-pareil.

A representative decision tree further supported that samples close to potential yield (i.e., yield gap > 0.90) were associated with mature orchards (i.e., age > 5) and when winter T_mean_ < 10.22°C, and April SRAD > 478.8 W m^–2^ (*n* = 223) (Figure 5). The largest yield gap nodes, e.g., with a normalized yield of 0.27 (*n* = 596), were found among mature orchards, and when winter T_mean_ was greater than 10.22°C and mean June daily T_max_ was lower than 34°C; another grouping of plots with large yield gaps (0.38, *n* = 520), were associated with young orchards, winter T_mean_ lower than 10.22°C, and June T_max_ lower than 32.19°C.

### Determinants for Light Interception

**FIGURE 6:**
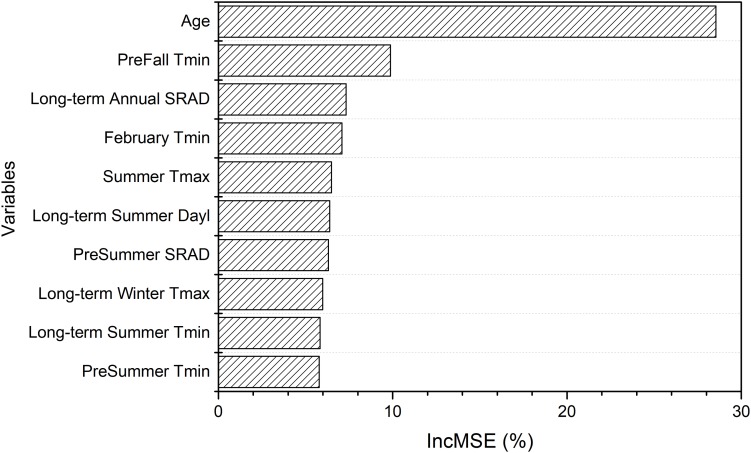
Variable importance for driving light interception derived from the random forest model using field variables and full meteorological variables.

**FIGURE 7:**
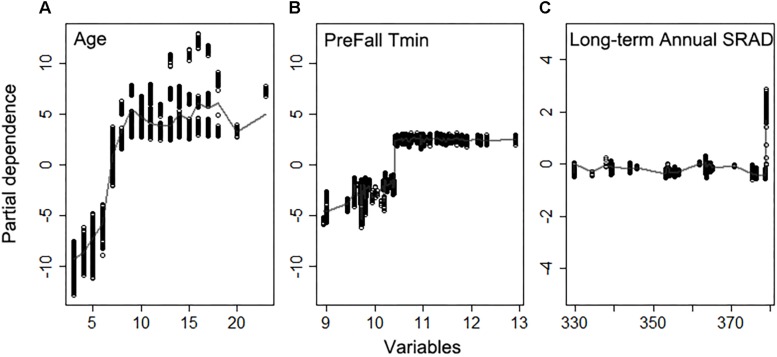
Partial dependence of the light interception on top three important variables **(A)** Age, **(B)** daily minimum temperature averaged previous Fall, **(C)** long-term annual mean shortwave radiation flux density (SRAD) for Non-pareil, ordered by variable importance.

The random forest model explained 82% of variation in light interception (Supplementary Figure S3), for Non-pareil (*n* = 5581), when using field based orchard characteristics and full set of meteorological variables as input. Age was the most important variable in determining light interception as expected, according to variable importance (Figure 6), and as shown by the high correlation (*r* = 0.63, *p* < 0.001) across all samples. The partial dependence plots further showed that light interception increased significantly with tree age until 7 years old and then plateaued (Figure 7). Mean Fall daily T_*min*_ in previous year, long-term mean annual SRAD, February T_*min*_, summer T_max_, and long-term summer Dayl also affected current year light interception. Fall T_*min*_ lower than 10.5°C (Figure 7B) and long-term annual mean SRAD lower than 380 W m^–2^ (Figure 7B and Supplementary Figure S5A) reduced light interception. We also found that other long-term mean climatic variables such as summer Dayl, mean winter daily T_max_, mean summer daily T_*min*_ had an important role, probably because they affected the general tree growth.

**FIGURE 8:**
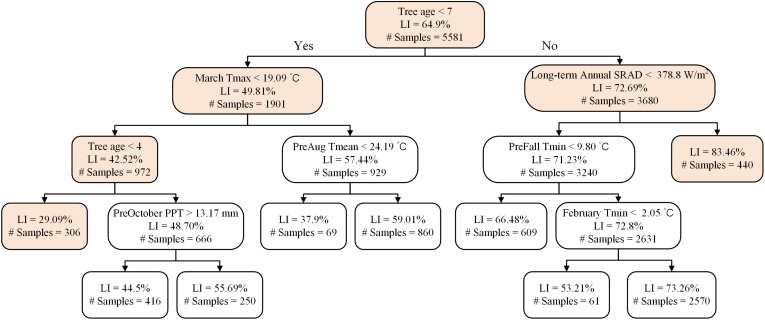
A representative decision tree on the determinants of light interception for Non-pareil.

A representative decision tree further revealed that light interception in trees < 7 years old was influenced by a different set of determinant variables than trees older than 7 years. In trees younger than 7 years the lowest light interception nodes were associated with mean March daily T_max_ < 19.1°C (Figure 8). In orchards > 7 years old, long-term annual mean SRAD > 378.8 W m^–2^ (Figure 8), and the majority of them were distributed in middle to southern Central Valley (Supplementary Figure S5B). For young orchards, the highest LI (59%) were those samples distributed from norther to middle, and southern Central Valley over various years (Supplementary Figure S6A). For mature orchards, the node with the lowest LI (53%) were 2013 samples clustered in the middle Central Valley (Supplementary Figure S6B).

### Overall Yield Prediction and Determinants

**FIGURE 9:**
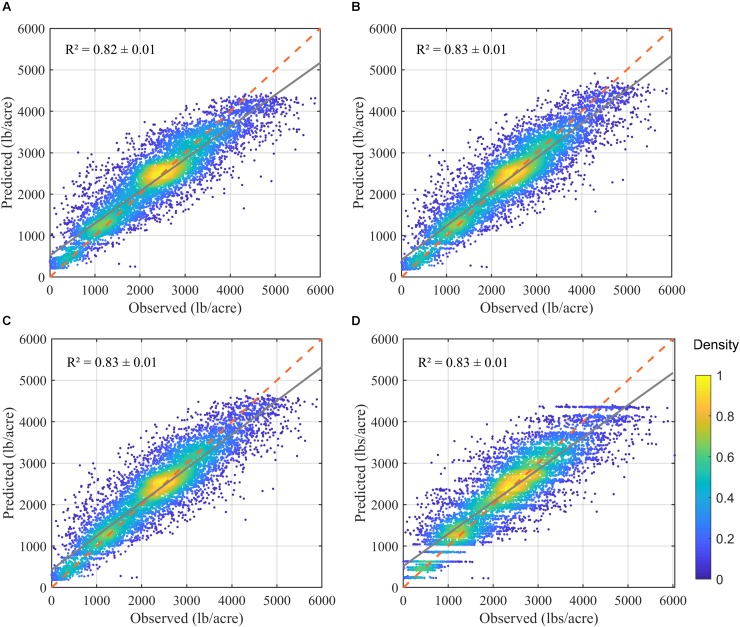
Comparison of the predicted vs. reported yield, based on the one realization of cross validation, from four separate random forest models: **(A)** using only biological variables; **(B)** using biological variables and full meteorological variables; and **(C)** using biological variables and selected meteorological variables; and **(D)** using full model but excluding light interception. Red dashed line denotes the 1-to-1 line, and the gray solid line denotes the linear regression trend.

The prediction results showed that all models were able to explain more than 78% of yield variation (Figure 9), much higher than the linear yield prediction based only on field measured light interception (*R*^2^ = 0.36), and the RF-based prediction using field measured light interception and orchard age (*R*^2^ = 0.60). For example, when adding other orchard characteristics such as age and location (i.e., latitude and longitude), model (A) had a *R*^2^ of 0.79 ± 0.01, RMSE of 530.64 ± 11.77 lbs/acre, and RPIQ of 3.12 ± 0.09, based on the random forest modeling with a sixfold cross validation. By further adding the whole suite of meteorological variables, the full model achieved the more robust and higher accuracy, as shown by higher *R*^2^ (0.82 ± 0.01), lower RMSE (480 ± 9 lbs/acre) and RPIQ (3.45 ± 0.17). After removing highly correlated meteorological variables, the reduced model with selected meteorological variables (Supplementary Figure S7 and Supplementary Table S2) had a similar accuracy with that of full model (Table 2).

**TABLE 2:** Yield prediction performance of random forest models driven by three sets of independent variables, based on the sixfold cross validation.

Models	RMSE (lbs/acres)	*R*^2^	RPIQ
A. Biological variables (including field measurements of light interception)	530.6 ± 11.8	0.79 ± 0.01	3.12 ± 0.09
B. Full set of biological and meteorological variables	479.5 ± 9.3	0.82 ± 0.01	3.45 ± 0.17
C. Full model but with selected meteorological variables	476.9 ± 6.6	0.82 ± 0.01	3.50 ± 0.15
D. Full model but excluding light interception	536.7 ± 9.6	0.78 ± 0.01	3.08 ± 0.09

When excluding light interception, the overall orchard characteristics (like location and age, tree density) and environmental variables (Model D) could explain 78% of yield variation across samples, similar to the model (A) which uses all orchard characteristics plus tree level light interception.

**FIGURE 10:**
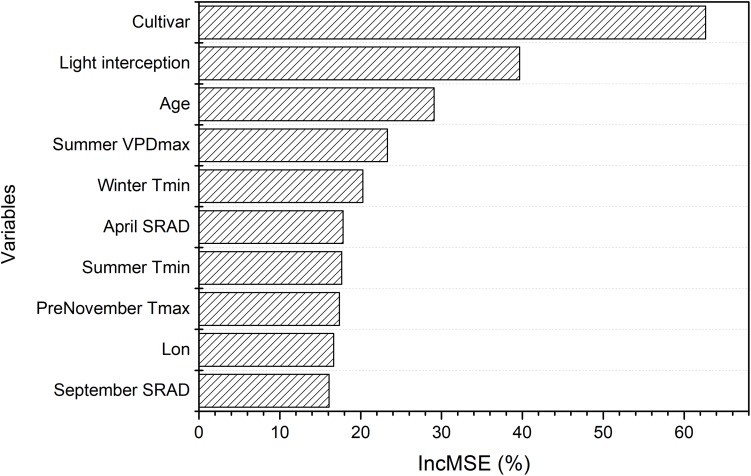
Variable importance derived from the random forest model of overall yield using field variables and selected meteorological variables, which is measured by the increase in mean-square-error (IncMSE) of predictions estimated when excluding each variable.

Based on the model with field biological and selected meteorological variables, we found that cultivar, light interception, and age were most important in determining overall almond yield (Figure 10). The key meteorological variables that ranked relatively important were mean summer daily VPD_max_, mean winter daily T_*min*_, April SRAD, and summer T_mean_ (Figure 10).

**FIGURE 11:**
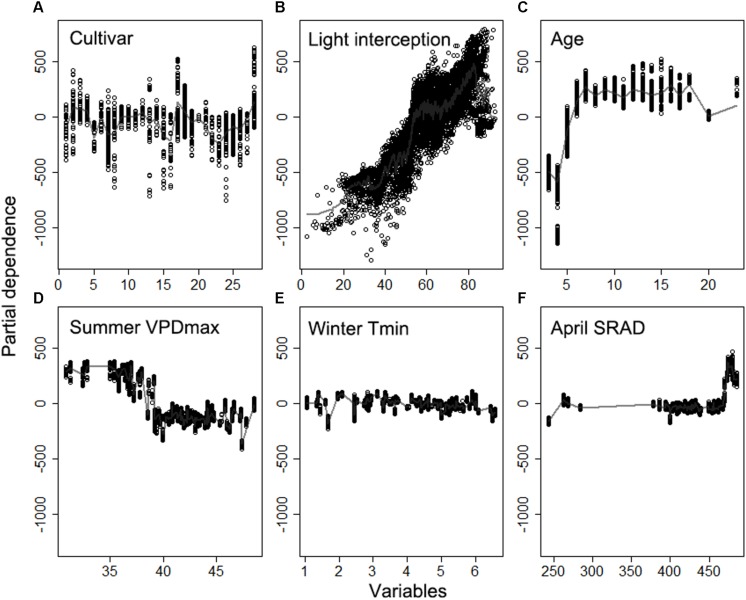
Partial dependence of the yield on top six important variables **(A)** cultivar, **(B)** light interception, **(C)** Age, **(D)** daily maximum vapor pressure deficit (VPD) averaged over summer, **(E)** daily minimum temperature averaged winter, **(F)** daily shortwave radiation flux density (SRAD) averaged over April, derived from the model using biological variables and selected meteorological variables.

The partial dependence analysis further showed yield difference across different almond cultivars (Figure 11A). Among the most popular almond cultivars, Aldrich (Cultivar ID: 2), Monterey (Cultivar ID: 17), and Non-pareil (Cultivar ID: 18) had higher yields than Butte (Cultivar ID: 5) and Carmel (Cultivar ID: 7), with everything else being equal. Yield increased linearly with light interception, but dropped rapidly when the light interception was higher than approximately 82% (Figure 11B). Tree age was identified to play an important role mostly during the young stage (i.e., 1–6 years) of almond growth (Figure 11C); the impact from tree ages was quite stable after reaching the maturity, but yield decreased for plots over 20 years of age. The contribution from April SRAD to the yield kept stable from 250 to 470 Wm^–2^, but rapidly increased after that threshold. In contrast, Mean summer daily VPD_max_ limited the yield when it was higher than 40 hPa (Figure 11D). The additional meteorological variables such as mean winter daily T_*min*_ did have a slightly negative impact on the yield variation (Figures 11E,F).

Among mature orchards only, (i.e., tree ages from 7 to 18) (*n* = 4337), variable importance and partial dependence plots showed that light interception was the dominant control on the yield for mature almond trees (Supplementary Figures S8, S9), the yield varied considerably across different cultivars. Almond cultivars with Monterey (2601 ± 458 lbs/acre) (*n* = 191) and Non-pareil (2401 ± 1152 lbs/acre) (*n* = 3293) were more productive than others (2052 ± 1110 lbs/acre, Supplementary Figure S10). The identified impacts from other meteorological variables were similar to those derived from the scenario using all almond samples (Supplementary Figure S9).

## Conclusion and Discussion

Tree crops have rather complex processes in terms of nut production, involving physiology of tree growth, flowering phenology, bee activity, and etc (Connell, 2000; Zarate-Valdez et al., 2015; Tombesi et al., 2017; Chen et al., 2019a, b). A large dataset across the environmental gradients coupled with a more advanced data analytics, such as artificial intelligence (AI) including machine learning (ML) algorithms, are needed to understand the constraints on the yield gap at the plot and field scales (National Academies of Sciences Engineering Medicine, 2019). This study made use of a unique dataset of field measurements of light interception and almond yield records in California’s almond orchards. We used random forest, a widely used ML approach, for interpreting and predicting the variations almond nut production. Our modeling experiments showed that the full random forest model explained about 82% (±1%) of yield variation using a sixfold cross validation, with a RMSE of 480 ± 9 lbs/acre). The RF-based prediction using only field measured light interception and orchard age (*R*^2^ = 0.60); when excluding light interception, the overall orchard characteristics (like location and age, tree density) and environmental variables could still explain 78% of yield variation across samples. Cultivar, light interception, and age were most important in determining overall almond yield. Various climate variables were also found to play important roles in yield variation.

Both seasonal weather conditions during the current year and the previous year were found to affect the plant physiology and thus nut production from year to year at the field scale. Long term climate, on the other hand, determines the spatial variation in the almond yield at the regional scale. Our results showed that, at a given level of light interception, the departure of the actual almond nut production from the potential yield varied significantly, driven mostly by temperature in June and winter, mean summer daily VPD_max_, and incoming solar radiation (SRAD) in addition to tree age. Warmer winter, e.g., limited the yield for the mature orchards from reaching the maximum yield. On the other hand, light interception fraction was found higher for mature sites with higher long term mean SRAD and lowest light interception for younger orchards and when March maximum temperature was lower than 19°C. For the overall almond yield, we also found that summer VPD_max_ limited the yield when it was beyond 40 hPa and warmer daily T_*min*_ also reduced the yield.

Further studies are needed to examine the stressors of extreme weather such as heatwaves on plant growth. We did find that the number of extreme hot days on the nut production had a negative impact, for example, extreme hot days in June either in preceding year or concurrent year had a considerable negative impact on yield (*r* = −0.31 and *r* = −0.21, Supplementary Figures S1, S7). However, when putting all other variables together, they didn’t show as top six environmental controls, probably because these heat threats could be partially reflected by other monthly climatic variables such as VPD and temperature.

Our results showed that the light interception was found as the predominant control for the almond yield. Overall the almond yield was highly dependent on light interception, e.g., one percent of increase in light interception led to an increase of 57.9 lbs/acre in the potential yield. The mobile platform (MLB) has been used to measure the light interception at the tree and plot level. Recent advances in UAV technology makes it possible to measure the energy reflected by plants at the meter or sub-meter scale (Johansen et al., 2018; Tewes and Schellberg, 2018), and estimate the plant biomass (Bendig et al., 2015; Liu et al., 2019), therefore providing another cost-effective way to map the light interception across the field scale. Moreover, satellite observations with higher spatial and temporal resolutions have been increasing in recent years, such as at 3m by PlanetScope. The optical observations at the RGB and NIR have long been used to monitor plant growth and photosynthesis (Zhang et al., 2003; Chen et al., 2019a). An important next step is to calibrate the relationship between the field measured light interception with the optical remote sensing observations from UAVs and drones, and then map the light interception at a larger scale.

In addition to its impact on plant growth, weather condition also affects the timing and intensity of bloom and bee activity in February and March, and therefore the nut production later in the season. The bloom information derived from high resolution remote sensing observations (Chen et al., 2019b) can be integrated into the yield modeling. Yield is also largely impacted by growers’ management practices including irrigation, nutrient, and canopy management such as pruning, weed management, and pests and disease control. The development of a large consistent database for location specific historic yield, orchard characteristics including the row orientation, and management history, is critical for future studies.

The machine learning approaches are expected to enhance both the explanatory power and the predictive capability, by bringing various big datasets together. A data-driven yield model based on advanced machine learning analytics, will allow researchers to query the causes and effects of location and year on productivity and to test current theories of the determinant of yields, a critical step in the development of improved sustainability practices. The prediction capability of the yield response to weather and climate, as shown by this study, is also expected to inform growers to adapt their management practices for plant protection under changing climate.

## Data Availability Statement

Publicly available datasets were generated in this study. This data can be found here: http://www.prism.oregonstate.edu/ and https://daymet.ornl.gov/.

## Author Contributions

YJ and PB conceived the project idea. BL collected the field data. BC compiled all the rest of spatial data, and built the models. BC and YJ performed the data analysis and wrote the manuscript. All authors reviewed and edited the manuscript, and agreed with the submission.

## Conflict of Interest

The authors declare that the research was conducted in the absence of any commercial or financial relationships that could be construed as a potential conflict of interest.

## Footnotes

1 https://cran.r-project.org/web/packages/randomForest/

2 https://cran.r-project.org/web/packages/rpart
