# Supplementary material for: Advancing Agricultural Production With Machine Learning Analytics: Yield Determinants for California’s Almond Orchards
Source: Front Plant Sci. 2020 Mar 13;11:290. doi: 10.3389/fpls.2020.00290 (PMC7082403; doi:10.3389/fpls.2020.00290)
Supplement: Supplementary file 1 [file Data_Sheet_1.pdf]

Supplementary Material for

Advancing Agricultural Production with Machine Learning Analytics: Yield Determinants for California's Almond Orchards

Yufang Jin[1*], Bin Chen[1], Bruce Lampinen[2], and Patrick Brown[2]

[1]Department of Land, Air and Water Resources, University of California, Davis, CA 95616, USA

[2]Department of Plant Sciences, University of California, Davis, CA 95616, USA

*** Correspondence:**
Yufang Jin
yujin@ucdavis.edu

Supplementary Figures

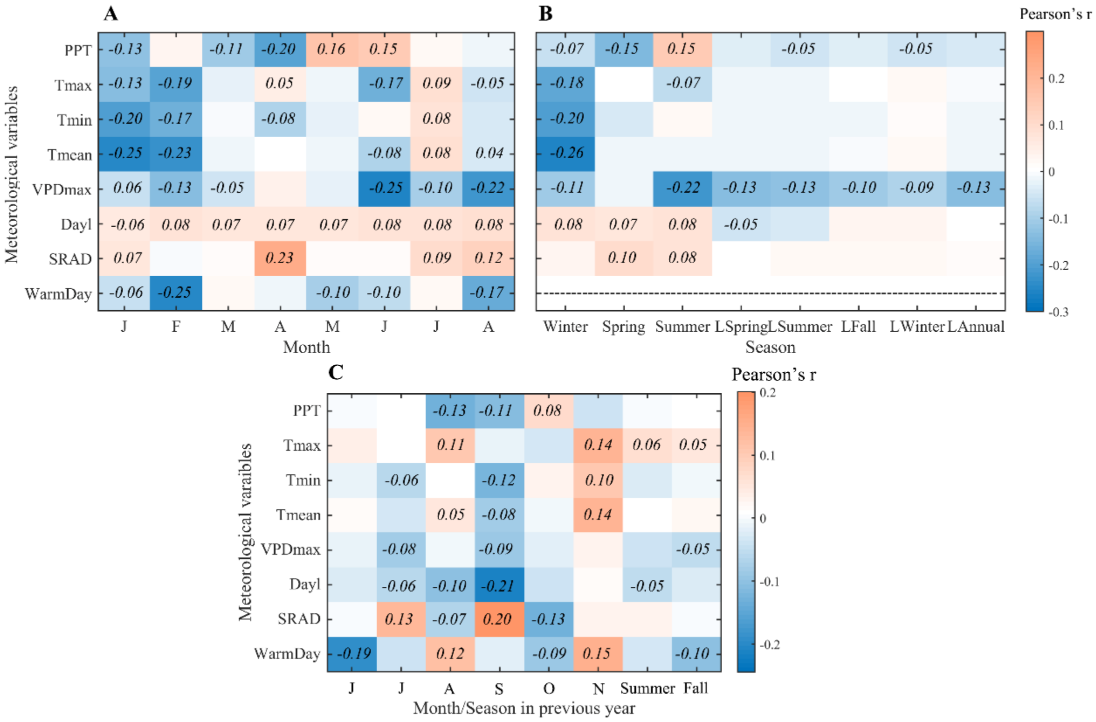

Supplementary Figure 1. The correlation between the normalized yield from Nonpareil samples (n = 5581) and (A) monthly and (B) seasonal meteorological variables in current year and (C) in preceding year. Note that the italic Pearson's r represents the correlation is statistically significant with a p-value < 0.001.

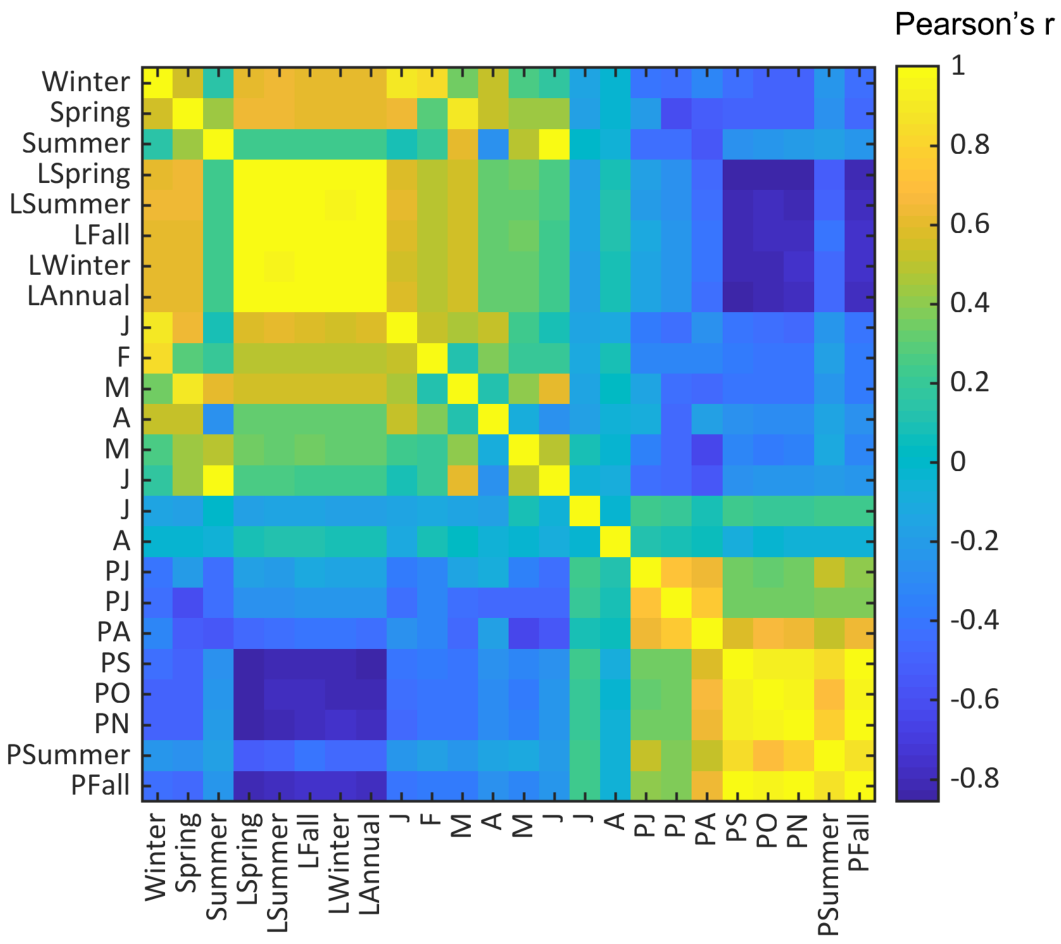

Supplementary Figure 2. Cross-correlation of variables from different time periods, taking precipitation as an example.

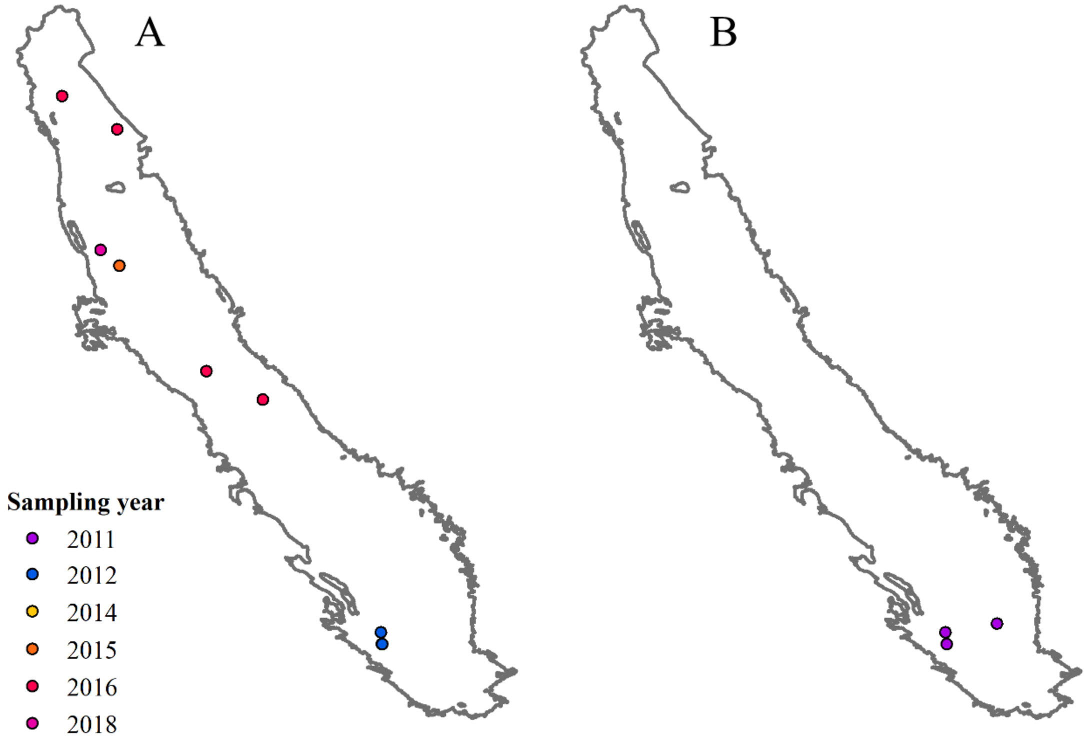

Supplementary Figure 3. Spatiotemporal distribution of samples with (A) largest yield gaps (N = 596) and (B) lowest yield gaps (N = 223).

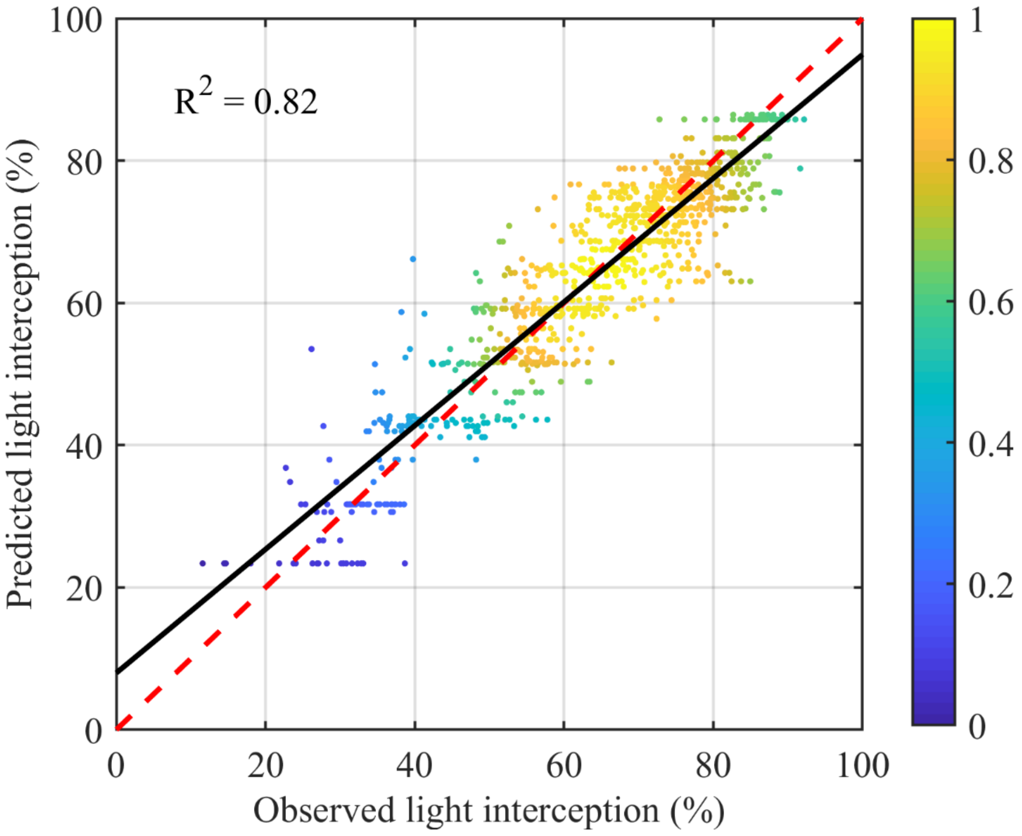

Supplementary Figure 4. Comparison of the observed and predicted light interception using biological variables and full climatic variables, based on the random forest model.

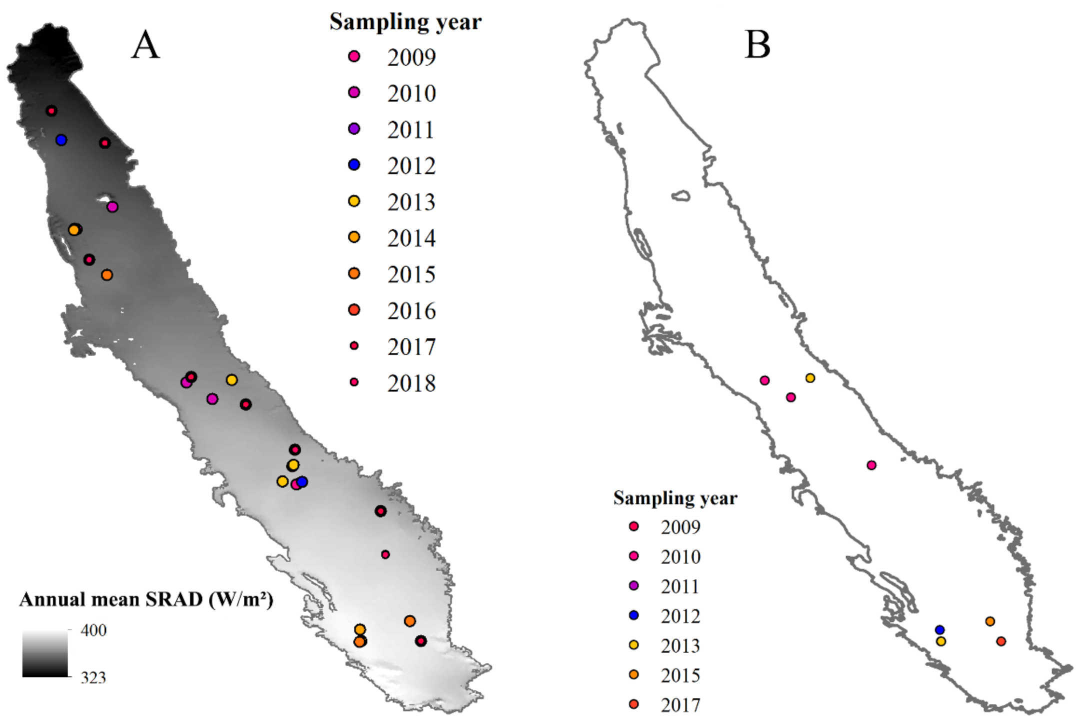

Supplementary Figure 5. Spatial map of (A) 10-year annual mean SRAD from 2009-2018 with almond sites colored by sampling years, and (B) almond sites with higher light interceptions (N = 440).

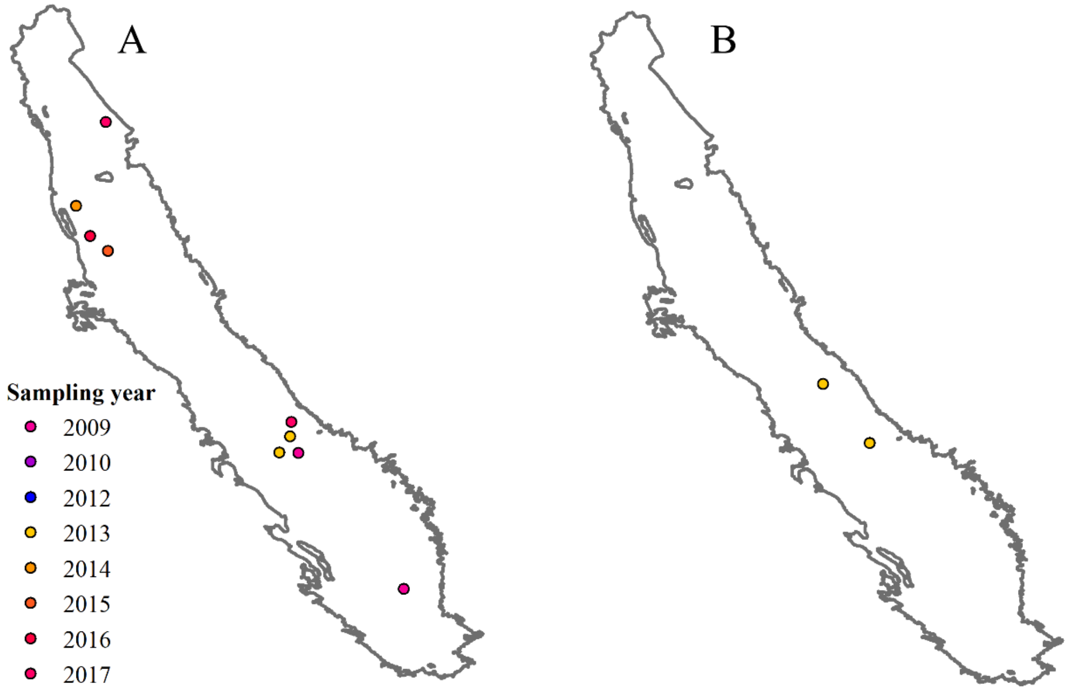

Supplementary Figure 6. Spatiotemporal distribution of samples with (A) higher light interceptions for young orchards (N = 860) and (B) lower light interceptions for mature orchards (N = 61).

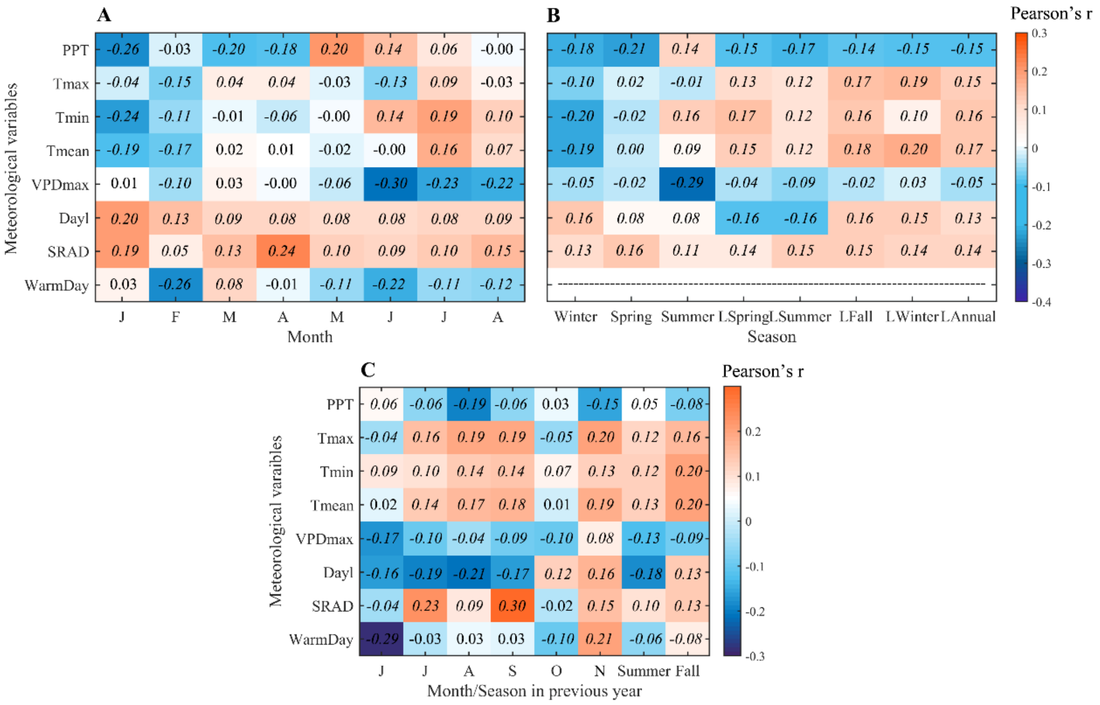

Supplementary Figure 7. The correlation between the actual yield from all almond samples (n=7864) and (A) monthly and (B) seasonal meteorological variables in current year and (C) in preceding year. Note that the italic Pearson's r represents the correlation is statistically significant with a p-value < 0.001.

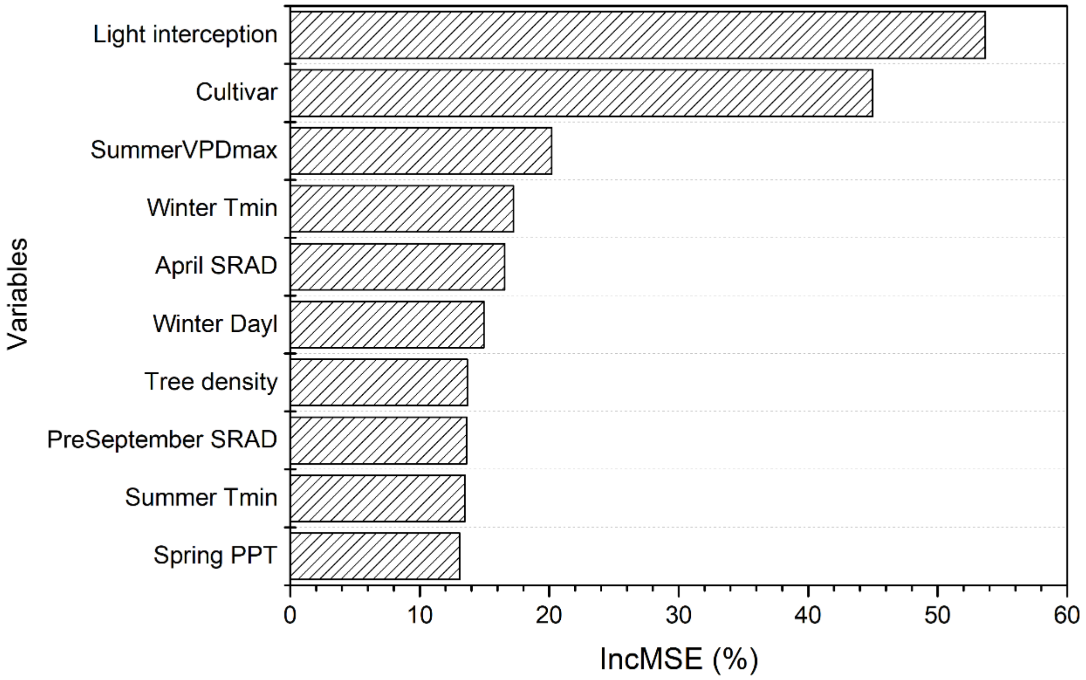

Supplementary Figure 8. Variable importance derived from the random forest model using field variables and selected meteorological variables.

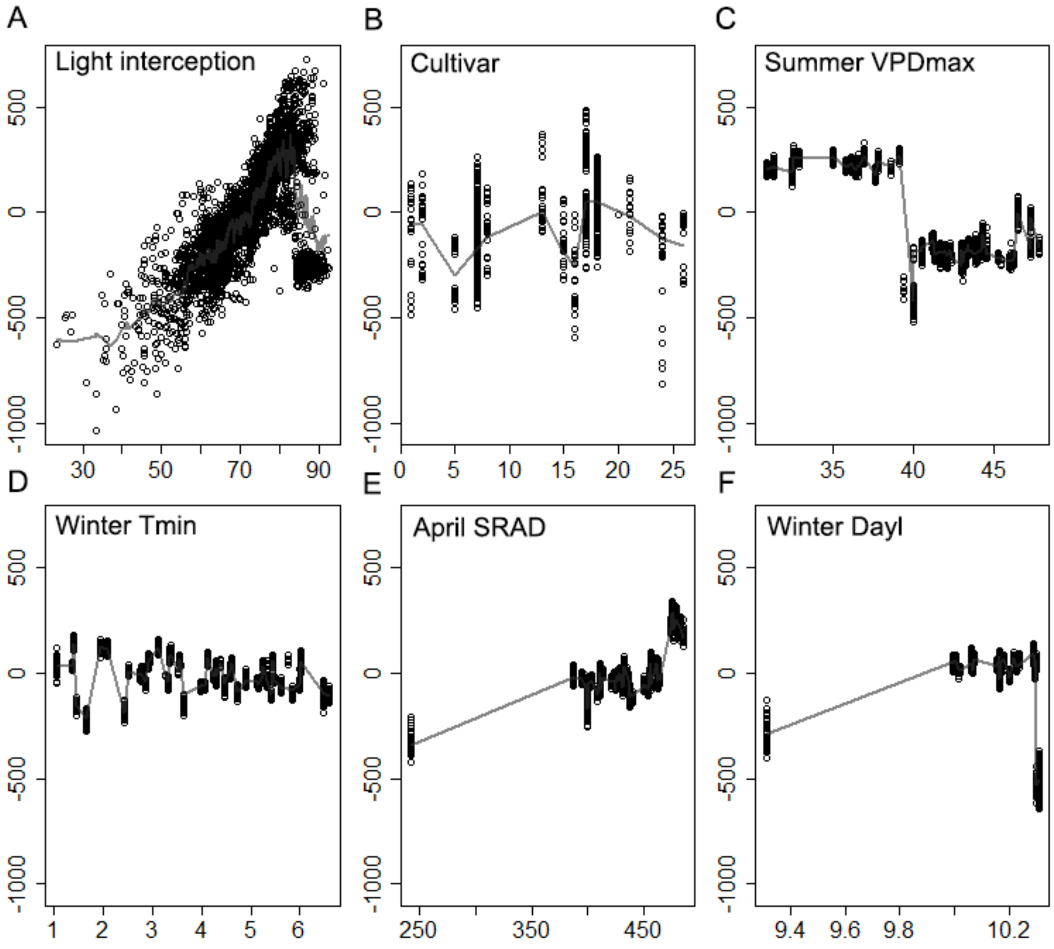

Supplementary Figure 9. Partial dependence of yield on different variables using mature orchards with age from 7 to 18 years (n = 4337).

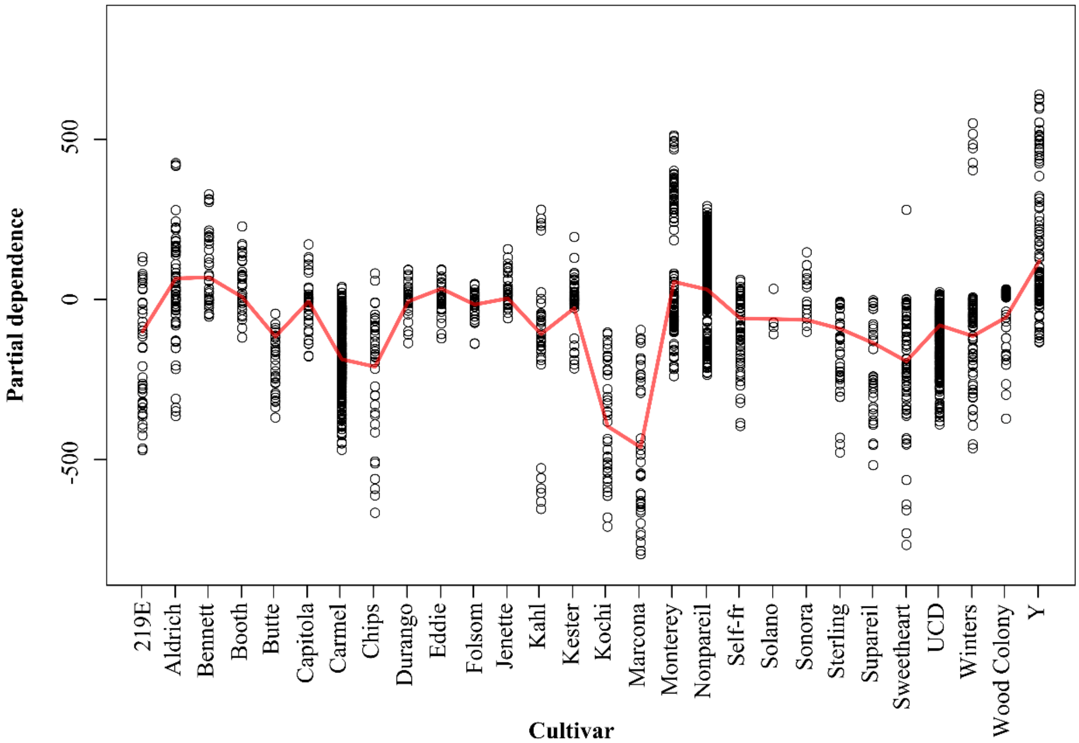

Supplementary Figure 10. Partial dependence of almond yield on different cultivars using mature orchards with age from 7 to 18.

Supplementary Table 1. Selected variables for modelling the determinants on yield gaps

**Category**	**Specific variables**
Precipitation	Spring PPT, Summer PPT
Temperature	Winter Tmean, mean June daily Tmax,
Vapor Pressure Deficit	Mean summer daily VPDmax
Daylight duration	Previous September Dayl
Shortwave Radiation Flux Density	April SRAD, Previous September SRAD
Number of Warm Day	February WarmDay, August WarmDay, Previous June WarmDay

Supplementary Table 2. Selected variables for modelling the determinants on the yield variation

**Category**	**Specific variables**
Precipitation	January PPT, Spring PPT, Long-term Summer PPT
Temperature	Previous mean November daily Tmax, mean winter Tmin, mean summer daily Tmin
Vapor Pressure Deficit	Mean summer daily VPDmax
Daylight duration	Winter Dayl
Shortwave Radiation Flux Density	April SRAD, Previous September SRAD
Number of Warm Day	February WarmDay, Preceding June WarmDay
